# How Should We Prepare Our Pharmacist Preceptors? Design, Development and Implementation of a Training Program in a Regional Australian University

**DOI:** 10.3390/pharmacy11050148

**Published:** 2023-09-17

**Authors:** Gillian J. Knott, Martina F. Mylrea, Beverley D. Glass

**Affiliations:** Pharmacy, College of Medicine and Dentistry, James Cook University, Townsville 4811, Australia

**Keywords:** experiential education, preceptor role, preceptor support, educator, mentor, assessor, participatory approach

## Abstract

Increased experiential learning within pharmacy education has highlighted the need for quality preceptors, leading to a recent focus on their development and training. This study describes the design, development and implementation of a pharmacist preceptor training program in a regional Australian University. The design of this program utilized the results of a previously conducted scoping review of preceptor training programs and a comprehensive preceptor training needs analysis. In addition, participation was sought from an expert advisory group, comprising preceptors and pharmacy professional organizations. Program materials were developed and transferred onto a suitable online learning platform for implementation. Post-training program evaluation will follow Kirkpatrick’s model of training evaluation criteria, gaining feedback from both preceptors and students. The four flexible online modules that were designed are aligned with the main preceptor roles: role model, educator, mentor and assessor. The program also included a small-group online interactive networking session and an online discussion forum. Following implementation, 28 pharmacist preceptors completed the training. Preliminary feedback on the training program was extremely positive, particularly to the interactive networking activity. Future programs will continue to be adapted to reflect stakeholder feedback and optimize student placement experiences.

## 1. Introduction

Preceptors play a vital role in pharmacy education by teaching and mentoring student and intern pharmacists in practice settings as they develop their professional knowledge and skills. Globally, the focus on experiential learning within pharmacy education has highlighted the increasing role of pharmacist preceptors and the need for preceptor training and development to ensure the provision of quality teaching. Training and support for pharmacist preceptors is thus regarded as essential and considered to be the professional responsibility of the university. The Australian Pharmacy Council (APC) Accreditation Standards for Pharmacy Programs require all professionals who are involved in assessing students or interns to be ‘suitably qualified, experienced and prepared for the role, and ‘provided with appropriate guidance and support’ [1]. In the United States (US), the Accreditation Council for Pharmacy Education (ACPE) Standards require that the college or school promotes the professional development of preceptors in line with their educational responsibilities [2]. There remains, however, a lack of in-depth guidance to inform the design, delivery and ideal content of professional development programs for preceptors [1,2,3,4,5].

Two global reviews of preceptor training programs revealed limited documented examples of training programs and a wide variation in the design, delivery and content of these programs [6,7]. Similarly, a study investigating preceptor orientation and development programs across various institutions in the United States found significant variation, with not all programs meeting the minimum expectations for preceptor orientation as outlined by the ACPE [2,4]. Preceptors supervise students in both hospital and community pharmacy, but also across a range of other less common or more specialized placement sites, such as general practitioner (GP) practices, specialist hospital clinics, industry and professional organizations. It is therefore not surprising that preceptor roles and responsibilities vary widely, which in part explains the diversity of training programs available [8]. While it is recognized that programs should preferably be tailored to suit the individual school or institution, it is also thought to be important that there is some standardization of key program elements such as preceptor orientation and student assessment and evaluation. Programs should be structured and evidence-based, but also provide flexibility for preceptors, who as clinical teachers, practice in a busy and often complex working environment [4,6,8]. The majority of preceptors are not remunerated for this role [4] which provides a further challenge to undertaking training.

Recent interest has focused on preceptor competencies to provide an underpinning structural framework for preceptor development, promoting consistency of preceptor performance and potentially enhancing the student learning experience. Preceptor competencies are closely associated with a range of ideal preceptor roles, skills and attributes, with their identification being necessary to inform the development of competency standards. Several studies have investigated the ideal skills and attributes of pharmacist preceptors from the perspective of both preceptors and students [9,10,11,12,13], with professional competency frameworks for pharmacists or other health professional preceptors under consideration in many countries [14,15,16].

In addition to competency standards and adherence to the requirements of professional accreditation bodies, the design of a training program for preceptors needs to consider available resources, the nature of the precepting environment, the background knowledge of the preceptor, their specific roles and responsibilities and their individual training needs [5,7,17]. With this in mind, the aim of this study was to design, develop and implement a pharmacist preceptor training program, tailored to meet the needs of community pharmacist preceptors at James Cook University (JCU).

## 2. Materials and Methods

### 2.1. Study Setting—James Cook University (BPharm (Hons))

Experiential learning provides the opportunity for students to link learned theory to the realities of pharmacy practice and is considered by the JCU program as an integral component of their curriculum, with students undertaking 600 h of placement during their degree, under the supervision of a pharmacist preceptor. JCU is a regional university with a focus on rural and remote practice; therefore, preceptors and placement sites are geographically distributed across the state of Queensland, covering an area of approximately 80,000 square kilometres. Placement blocks vary in length, ranging from two to six weeks, with all students required to attend at least one rural placement. This training program was designed to prepare pharmacists for their role as student preceptors, with a particular focus on community pharmacy preceptors, who represent about 70% of JCU’s cohort of volunteer preceptors.

### 2.2. Instructional Design Model

**Figure 1:**
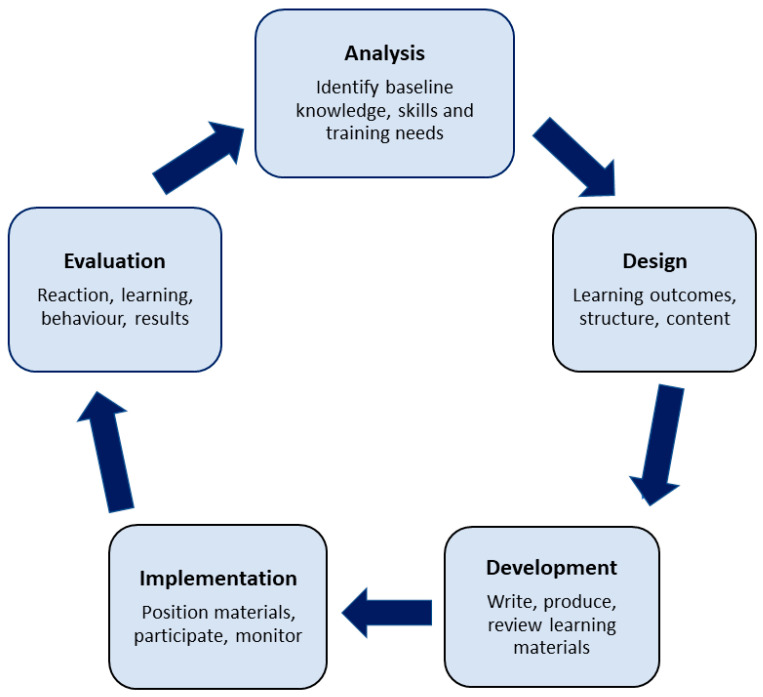
ADDIE model for instructional design. (Adapted from [18]).

The 5-point ADDIE (Analysis, Design, Development, Implementation, Evaluation) instructional design model (Figure 1), which has been found to be particularly relevant for online and distance education [18], was used to inform the overall design of the training program. This model consists of five phases: Analysis, Design, Development, Implementation and Evaluation. In the analysis phase, the baseline knowledge and skills of the learner (preceptors) and their training needs are identified. This information is then used in the design phase to create learning outcomes, to choose the most effective and convenient learning tools and appropriate program content, and to decide upon the best learning platform. The design phase is followed by the development phase, where the learning materials are written, produced and reviewed within the online platform. Once the program has been finalized, it is implemented, with the trainees (preceptors) participating in the program, with regular monitoring and minor adjustments made if needed. The final phase, after participants have completed the program, is to choose an appropriate method of evaluation to ensure that the program has met the learning outcomes and to identify areas for improvement for future programs.

### 2.3. Analysis and Program Design

**Figure 2:**
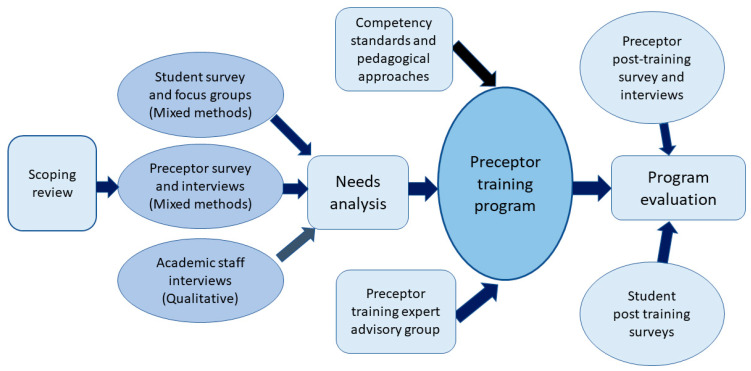
Overview of approach to inform the design of a preceptor training program.

A participatory approach was employed to inform the design of this training program, enabling the program to be tailored to those directly involved in experiential teaching and learning. This approach included a needs analysis of preceptors, academic staff and students, using mixed methods, and collaboration with an expert advisory group. Figure 2 provides an overview of the approach taken to inform the design of the program. The needs analysis which informed this program has been reported in previous publications [9,10], with this article focusing on the design, development and implementation of the training program. Furthermore, as the program evaluation is in the preliminary stages and therefore not currently completed, evaluation results will not be discussed at this time, being the subject of a future publication.

Following a scoping review of preceptor training [6], both qualitative and quantitative methods were employed in an explanatory sequential needs analysis study. The purpose of this study was to identify the roles and associated skills and attributes of the ideal preceptor as well as their training and support requirements from the perspective of all key stakeholders in experiential learning. Preceptors and academic staff were also asked to indicate their preferences regarding structure and content of a training program, and their perceptions on key enablers and barriers to training, which were considered in the program design [9,10]. 

The curriculum framework for this program was based on a preceptor training program developed by Condrey [19], which outlined four roles of the preceptor: role model, educator, mentor, and assessor. The Pharmaceutical Society of Australia’s (PSA) National Competency Standards Framework for Pharmacists [20] was examined and linked where relevant to the program content. Appropriate pedagogical approaches for adult learners in the online environment also informed the educational design of the training program [2,21,22,23,24,25,26].

After the preparation of a draft curriculum for training, the research team established an expert advisory group to provide oversight, especially in relation to the practice, and to contribute to the program design. Group members were chosen by purposive sampling and invited to participate in two online meetings, one prior to program development and one following the completion of the training program by preceptors. The advisory group included the three members of the research team (the authors), two members from key professional pharmacy bodies (PSA and APC) and two preceptor representatives, from both a rural and an urban JCU placement site. The research team are all academic staff members in pharmacy at JCU, with the principal researcher (GK) being an experienced pharmacist and academic, BG being a pharmacist with extensive academic and research experience and MM having a degree in education as well as significant academic experience. To minimize the risk of bias in the program evaluation, the two preceptors involved in the expert advisory group did not participate in the preceptor training program. Advisory group meetings were recorded and minutes were taken, which allowed the researchers to fully examine member comments and suggestions for program improvements.

Overall, advisory group members were complimentary of the proposed training program, in terms of both structure and content. Various issues were discussed, including student motivation, the challenges of providing feedback, preceptor competencies and linking preceptor training to career progression. Additional content topics suggested by group members included leadership and teamwork, an expansion of cultural considerations, reflective practice and different student learning styles. Based on feedback from group members, the preliminary draft of the training program was modified and further developed into its final form. A second advisory group meeting was held following completion of the program to report on the program implementation and to discuss any future improvements to the program.

### 2.4. Program Development and Implementation

The program materials were developed by the principal author and reviewed by the research team. A suitable platform for delivery was selected and prepared and program materials were built as modules within this learning platform. Program implementation was timed to coincide with the schedule of student placements, allowing preceptors to complete the full program prior to hosting their next student. A certificate of completion was provided to all preceptors who completed the program.

### 2.5. Program Evaluation

For quality assurance, the program will be evaluated, guided by Kirkpatrick’s framework model for evaluation of educational effectiveness, which consists of four levels of evaluation criteria: reaction, learning, behaviour and results. The reaction level measures the trainee’s perception of training, i.e., did the learner (the preceptor) enjoy the training. The learning level is a measure of the degree of learning achieved by the trainee, based on the learning outcomes., i.e., was the program informative to preceptors. The behaviour and results section are more difficult to measure and involve ascertaining the effect of the program on work habits and performance outcomes, i.e., did it improve precepting ability and confidence and lead to improved student learning [27].

This evaluation will involve a preceptor self-completion survey immediately following completion of the program, as well as preceptor post-training interviews, conducted shortly after a student has completed a placement in their community pharmacy. The preceptor post-training survey will address the reaction and learning levels of Kirkpatrick’s evaluation of educational effectiveness model. The post-training interviews will seek to identify any self-reported changes to precepting behaviour resulting from the training, thus addressing level 3 of Kirkpatrick’s model. To provide a balanced perspective, the program will also be evaluated from the viewpoint of the student by making use of the standard *Student evaluation of the preceptor* survey which is routinely conducted by JCU Pharmacy following each student placement. Student post-training surveys for trained preceptors will be compared to untrained preceptors, to identify any changes to precepting behaviour because of training, thus adding to the accuracy of the level 3 evaluation.

## 3. Results

### 3.1. Analysis and Program Design

The scoping review identified a range of preceptor training programs, for both hospital and community preceptors as well as residency preceptors. Modes of delivery of training included face-to-face classes and web-based modules, supplemented by a preceptor manual, assignments and recommended readings. While online training was convenient and often considered a necessity due to the geographic distribution of preceptors, face-to-face training was thought to better facilitate preceptor networking. Program flexibility was important to preceptors due to time and workload constraints. Areas of interest for training content included teaching skills, how to be a good role model, conducting assessment and the provision of student feedback. Various useful support measures were identified, including regular communication with preceptors, recognition of the work of preceptors and the provision of continuing professional development (CPD) credits for participation in training. Training program evaluations were mostly positive and based on post-training qualitative feedback from preceptors, although some programs conducted pre- and post-preceptor surveys or interviews, with only a small number of programs obtaining student feedback [6].

The preceptor training needs analysis reinforced the benefits of preceptor training and made recommendations that all preceptors undertake training. It was agreed that preceptors play many roles, with their key role being to link theory to practice. Important preceptor qualities include good communication and counselling skills, being a good role model, interest and enthusiasm about precepting and providing effective, and timely and constructive feedback. It was felt that training should be predominantly provided online with flexibility of training a key requirement. In addition to the online component, some form of interactive session was desirable, as well as a supplemental preceptor manual (which JCU already provides).

Training program content should include an overview of the university curriculum, preceptor and student expectations, teaching tips and strategies, conflict management and how to provide feedback. As support for preceptors, the university should communicate regularly and involve preceptors in current and future curriculum development. The most significant barrier to preceptor training was time, although lack of training and the voluntary nature of precepting were also considerations when choosing to become a preceptor. The main benefit of training was the delivery of a consistent approach to precepting, which was thought to lead to an improved student placement experience [9,10].

**Figure 3:**
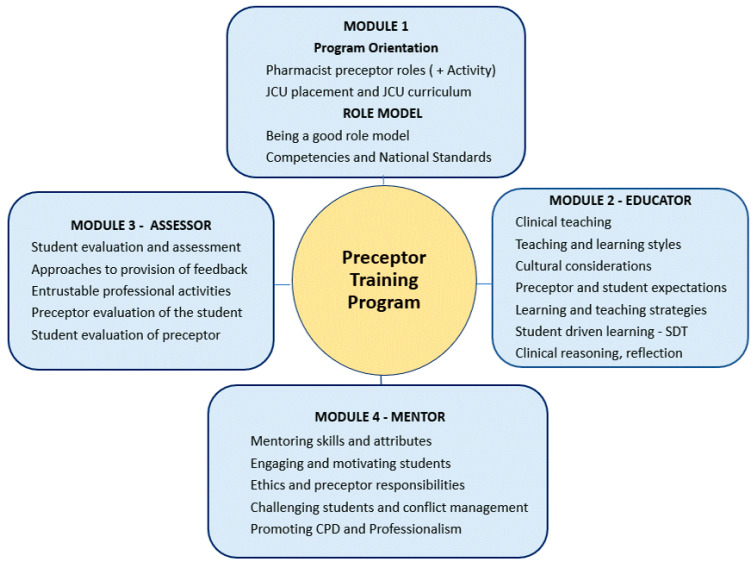
Overview of online modules for preceptor training program.

The decision to conduct an online program was influenced by practicality, with the geographic distribution of preceptors as well as time pressures being a major impediment to simultaneous face-to-face training. While face-to-face programs are relatively common in the United States and are often preferred due to their interactive nature, they can be a challenge in circumstances where there are limited resources and poor preceptor accessibility [5,17]. This approach was considered not to be feasible in the JCU context, and a blended online format was chosen, which included both online modules and an online interactive component. The program that was developed consists of four online modules, in addition to a small-group interactive preceptor networking session and an online discussion forum. Figure 3 provides an overview of the four modules, with an outline of the final program available in Supplementary Table S1. The chosen instructional design offers the required flexibility for preceptors as well as addressing the desire for interaction, as identified in the needs analysis.

The individual modules contain instructional materials which address the four main preceptor roles outlined in the Condrey program: role model, educator, mentor and assessor [19]. Although Condrey’s program was designed for registered nurse preceptors, the principles of effective precepting are similar across the health professions [14,15]. As a health professional, preceptors are an important role model for student to observe and follow. As educators, although much of the theoretical knowledge of students is learned at university, preceptors supplement this knowledge and link theory to practice. Preceptors also provide mentorship to pharmacy students, whereby they guide and support them as they develop their professional skills. In their role as an assessor, preceptors evaluate students and provide essential feedback, which allows students to grow and learn from their placement experiences [22,28].

While Condrey’s program included an online component for theory-based information and a four-hour face-to-face training class, the JCU program was conducted fully online and therefore these two components were integrated across the four modules. Whereas in Condrey’s program, face-to-face training concluded with small group and class discussions of case studies based on the four preceptor roles, in the JCU model, this session was conducted as a small group online synchronous case-based interactive session, following the completion of the online modules.

The module materials incorporate pedagogical approaches, and teaching and learning theories which are relevant to the experiential learning context. Preceptors are clinical educators who teach students in a practice environment. They are also adult learners; therefore, it was considered that andragogical teaching methods featuring a constructivist approach would be most applicable for preceptor training. The preceptors are introduced to a range of educational theories and concepts, such as deep and surface learning, intrinsic motivation, the VARK learning style classification, Miller’s pyramid for clinical teaching and assessment, and the ACPE preceptor teaching methods [2,21,22,23,25]. The overall learning outcomes for the modules are provided below:

- To identify and describe the roles of the pharmacist preceptor and their associated skills and attributes.
- To have a knowledge of the skills required to be a student role model and to promote the profession of pharmacy and the importance of lifelong learning.
- To recognize the different teaching and learning styles which may impact on the education of pharmacy students during placement and to be able to implement a variety of teaching strategies.
- To describe the principles of mentoring or coaching to motivate, guide and manage pharmacy students during their placements.
- To understand the principles of student assessment and strategies for the provision of student feedback in the clinical environment.

A welcome message and introductory materials are also included. Each module contains a set of individual learning outcomes, along with information relevant to the preceptor role, online activities, a range of brief supporting video clips, a list of references and a ten-item multiple choice quiz to provide immediate feedback and allow the preceptor to reflect on what they had learnt. The modules are designed to be flexible and able to be completed in the preceptor’s own time over a five-week period, with the estimated overall completion time for the modules being six hours. 

It was felt that for an optimal outcome, preceptors should be advised to complete all modules and their associated quiz prior to attending a 1.5 h online synchronous networking session. The networking session was designed to allow preceptors to interact with other preceptors in a small group, thus encouraging the sharing of information and ideas. A series of six case studies were prepared to promote discussion. Cases were linked back to various aspects of precepting which were covered in the four modules. This included pre-placement preparation, setting expectations, teaching styles, one-minute preceptor, student assessment and conflict management. As examples, one case provided a student scenario from which preceptors could use the one-minute preceptor framework to provide student education and feedback and one case presented a situation where a student is lacking motivation and behaving in an unprofessional manner, asking preceptors to discuss their response to the situation.

The decision to establish an optional online discussion forum within the organizational site was intended to facilitate further asynchronous communication between preceptors to share ideas and strategies for precepting at their own convenience.

### 3.2. Program Development and Implementation

An organizational site called the *JCU Pharmacy Preceptor Training Hub* was developed as part of the JCU Blackboard^®^ Learn educational platform, and with the help of a digital support assistant. As the already established educational platform for JCU, this was convenient and was able to be easily accessed at no cost to the preceptors. Program modules were prepared and loaded into this online organization site. An image of the preceptor training organizational site is provided in Supplementary Materials (Figure S1).

Following the preparation of the organizational site, a list of email addresses of current and past preceptors was obtained from the JCU pharmacy placements administrative officer. All 240 current and past preceptors were invited by email to participate in the program, with 62 pharmacist preceptors (25.8%) registering for the program. After completion of the four online modules, seven small-group online networking sessions were conducted with preceptors. A maximum number of nine preceptors per group was set, to ensure that there was ample opportunity for all group members to participate in the discussion. These sessions were facilitated by the main author, with each of the six case studies being presented and discussed among the group members. Opportunities for general discussion were also provided within the session. Preceptors who had registered for the program were also invited to contribute to the online discussion forum. Several comments were added to the forum by the facilitator to promote conversation.

Of the 62 registrants, 33 preceptors commenced the modules, and 28 preceptors (46.8% of registrants) completed the entire program and were therefore eligible to receive a certificate of completion.

### 3.3. Program Evaluation

Following participation in the program, and completion of all components, including the module quizzes and the preceptor networking session, preceptors will be invited to complete a 10 min post-training online feedback evaluation survey, developed using the Qualtrics^®^ survey platform (Qualtrics, Provo, UT, USA).

As a follow-up to the survey, a sample of preceptors will be invited to participate in a post-training interview, to be conducted shortly after hosting their first post-training placement student. The aim of the interviews will be to ascertain the outcomes of the training program in terms of changes in their knowledge and attitude towards precepting and the impact on their behaviour during the placement. Interviews will be conducted by telephone and audiotaped, with an estimated completion time of 15 to 20 min. Program evaluation will also be undertaken from the perspective of the students through the *Student Evaluation of the Preceptor* post-placement survey, which is routinely conducted online via Qualtrics after each placement experience. All student surveys in the year following the program will be collected, with the surveys of trained preceptors being compared to untrained preceptors, to identify any changes to preceptor evaluations following program participation. The post-training evaluation of this preceptor training program will be the subject of a future article.

## 4. Discussion

The goal of this training program is to prepare and support JCU pharmacist preceptors in their role as teachers and mentors of JCU students during experiential placements. The program design was informed by JCU preceptors, students, and academic staff, with additional input from key industry stakeholders. In line with stakeholder requirements, the program provided individualized training via a set of flexible online modules, together with a small group interactive online networking session. Curriculum content was tailored to suit the identified needs of JCU preceptors, and the program was evaluated from the perspective of both preceptors and students. This discussion will follow the five stage ADDIE Model of Instructional Design which guided program development.

A needs analysis is a commonly used method of identifying trainee requirements to develop an effective and evidence-based training program [29,30]. A range of preceptor needs analyses have informed the design of pharmacist preceptor training programs. Most studies used a single method of investigation, either quantitative surveys or qualitative interviews or focus groups, with many involving the preceptor only and some focusing more on ascertaining the views of faculty, program coordinators and educational experts, rather than preceptors [5,8,17,26,30,31,32]. The needs analysis conducted prior to the development of this program used a mixed methods approach, which provided both breadth and depth of data for analysis. The participatory approach also ensured that those who stood to benefit from this program, including preceptors, students, and academic staff, were consulted to inform the program design, allowing for tailoring of the program to the specific needs of JCU pharmacist preceptors.

Common modes of delivery in recent preceptor development programs have included electronic methods such as on-demand or live webinars, online modules, and face-to-face classes, with many programs choosing a blended delivery approach incorporating online and face-to-face methods [4,7,19]. This study, like many others, identified the importance of a flexible approach to training delivery, while also acknowledging that preceptors appreciate some form of live interaction with their peers [8,26,29,30].

From this study, important preceptor skills and qualities included good communication, being a professional role model, enthusiasm for precepting and being able to provide good feedback. This aligns well with the literature [12,13] and relevant competency standards [14,15]. Suggested content areas for training also correspond well with documented pharmacist preceptor training programs and include providing feedback and managing conflict, curriculum structure and requirements, setting learner expectations and teaching strategies [4,5,33].

The second phase of the ADDIE model focused on the design of the training program, which considered both the overall structure and the proposed curriculum. It was decided that for this program, an online format was most appropriate due to the wide geographical distribution of preceptors and placement sites. There has been some debate over the most effective way to teach online, with studies finding that while online learning is a valuable tool for teaching clinical skills, a blended approach to learning was preferred [4,5,34]. The range of potential web-based instructional strategies, e.g., webinars as well as increased internet accessibility and improved reliability have promoted the development of online training. There is evidence that online instructional training may now be as effective as live face-to-face training in promoting participant interaction and engagement [35].

The instructional design of this program considered the nature of the learners (the preceptors) who are clinical teachers facilitating student learning and skill development in the practice setting. Clinical teaching can be complex; while practicing in a dynamic working environment, preceptors must not only create a safe learning space for students, but also ensure that they are managing their workload and providing optimal patient care [22]. Pharmacist preceptors are also adult learners, with varying degrees of experience in their field of practice and generally a high level of internal motivation and independence. In this context, andragogical teaching methods featuring a constructivist approach, were felt to be most applicable for preceptor training. The andragogical or adult learning approach, originally formulated by Knowles, focuses more on understanding and the application of prior knowledge and problem solving rather than the learning of content and facts [22,25,26]. Constructivism postulates that learners construct knowledge by reflecting and building upon their previous knowledge and experience [22,24]. Using this pedagogical approach, tasks which represent authentic and relevant examples of experiential teaching were designed. These required the preceptors to reflect upon and apply their existing knowledge to critically analyse student scenarios in the precepting setting. It was recognized that methods for effective online learning may differ from conventional learning, with one study finding that video clips were the most useful feature in online learning and online readings were the least useful [34]. The principles of andragogy apply to both the preceptor and the pharmacy student, with Kolb’s experiential learning theory supporting the focus on the student learner as an active participant in the educational process [22,24].

Given that this program was designed to teach preceptors how to precept students, an understanding of both learning and teaching strategies needed to be included, with a range of educational theories, tools, and frameworks being introduced or integrated into the program. In the Educator module, preceptors were introduced to the VARK learning style classification, which describes four learning modes for students: visual, aural, read/write and kinaesthetic, reflecting the different ways in which students learn. Research has found that by identifying the preferred learning style of the student and adapting their teaching style to suit the student, learning can be improved [36]. It is well known that differences across generations can lead to conflict [37] and this module provided preceptors with some insight into the generational divide and how each generation can work together and appreciate their differences. Biggs outlined two types of learning, deep and surface learning. Deep learning is preferred, as it promotes a healthy attitude to learning and increased retention of knowledge. Being able to link theory or content to hands-on activities, which is an essential part of experiential placement, was regarded as a useful skill to encourage deep learning [23]. Motivation is also important for student learning. Self-Determination Theory (SDT) describes the three key needs for human motivation: Autonomy, Competence and Relatedness. SDT was introduced as an underlying concept for preceptors to encourage autonomy supportive teaching. This will promote intrinsic motivation and lead to a deepening of student interest, increased reflection and promotion of critical thinking skills [21].

The ACPE identify four main preceptor teaching methods: direct instruction, modelling, coaching and facilitating, which may be used by preceptors at various stages of student development to enhance learning and progress their professional skillset [2,38]. Because preceptors are often time poor, the introduction of the One-Minute Preceptor clinical teaching tool, which can be implemented for case-based scenarios in the practice, was considered an effective way of improving time efficiency for preceptors [22,25,33]. In terms of assessment, Miller’s Pyramid is a well-known framework for the development of clinical competence and a knowledge and understanding of this framework should assist preceptors in evaluating and assessing their students appropriately [22,24].

A range of pharmacist preceptor training programs have been developed in recent years, including small, site-specific programs, university-wide programs and large consortium-based programs [4,6,7]. They may have been designed for preceptors in general, or specifically for community or hospital pharmacy preceptors, preceptors of students, interns, or residents. In the United States, they may focus on preceptors involved in introductory pharmacy practice experiences (IPPEs) or advanced pharmacy practice experiences (APPEs), or both [32,39,40,41,42]. Very few programs have been developed specifically for community pharmacy preceptors, with most being delivered predominantly face to face [32,40,43]. A web-based preceptor training program was developed across three universities in Australia, consisting of five modules, with Module 1 being introductory and the remaining four modules focusing on the student, the preceptor, precepting challenges and problems, and putting theory into practice [44]. A program developed in the United States in 2012 through collaboration with two universities included four core 30 min web-based modules, which covered an introduction to the professional experience program, the role of the preceptor, developing your rotation and providing feedback. Additional modules for further preceptor development continued to be added to this program over time [39]. A consortium- based program across five United States institutions consisted of five one-hour online modules entitled *Professionalism, Structuring the student’s experience, Motivating the student in the clinical arena, Achieving synergy in clinical teaching* and *Assessment and evaluation* [41,45]. In the United States in 2004, a multi-university training program was developed for an academy of preceptors, which in addition to live training and a preceptor manual, included a CD-ROM containing 3 modules, with Module 1 outlining preceptor roles and responsibilities, Module 2 focusing on assessment and Module 3 covering the provision of drug and health related information [42]. An interprofessional program developed in Qatar in 2022 consisted of a 13- hour online program which included five modules: *Principles of learning and teaching, Planning for experiential learning, Teaching and instructional strategies, Student assessment and feedback* and *Communication skills for effective preceptorship and conflict resolution* [30]. On close examination, it was determined that while the division of content across modules varied between these documented programs, much of the overall content was similar, and aligned well with the JCU program.

One of the most desired requirements for a pharmacist preceptor training program is the provision of continuing professional development (CPD) credits for training. Since preceptors are time poor and frequently contribute in a voluntary capacity, they will often prioritise their time based on potential rewards. CPD credits for training are a feature of many current training programs and the integration of preceptor development tools into professional CPD portfolios may also encourage preceptor participation in training in the future [46,47].

Following the development of the course materials and review, the JCU online program was made accessible to participants, with 28 preceptors completing all components of the program. It was thought that this relatively low participation rate was due to the current high workload of community pharmacists in Australia, considering also the impact of the COVID-19 pandemic on workload and staff absences [48]. Of note, while 240 preceptors were invited to participate, only 95 of these (39.6%) were active preceptors, which is defined as having agreed to be available to host a student within the previous 12 months. Furthermore, while preceptor training is recommended by the APC, the voluntary nature of precepting poses difficulties in mandating preceptor participation. This is the experience for many current programs with low participation rates being commonly reported [4,42]. In O’Sullivan’s study on preceptor orientation and development programs in 2020, it was found that while most schools offered preceptor orientation and development at least annually, on average 25% of the school’s preceptors attended live preceptor development events [4]. A study in 2020 found that preceptors teaching more experiential placement students are more likely to participate in training. They suggested that advertising the program to coincide with common precepting schedules may improve preceptor participation rates [49]. This program was designed to provide an orientation and tailored training to JCU preceptors; however, it did not consider the experience of preceptors. Studies indicate that preceptors who have been supervising students for several years felt that training is currently geared towards novice preceptors and there is a need for further individualization of training to cater for more experienced preceptors [5,31,50].

Learning settings can be enriched through collaboration and interaction, with preceptors valuing opportunities to network and interact. [4,5,39]. While the online discussion forum included in this program was intended to promote interaction, the use of the forum was particularly low, again reflecting the current high workload of pharmacist preceptors and the optional requirement for participation. Low participation in the discussion forum may also have been the result of the compulsory requirement for attendance at the small group interactive online training session, which provided an alternative and more direct means of dialogue between preceptors.

Preliminary results from the post training evaluation survey indicate that preceptors were overall very positive about the program, particularly appreciating the online networking session. The full results of the post training evaluation will be the subject of a future article.

Limitations of this study include the low number of preceptor training program completions, which was thought to be due to the already high workload of pharmacist preceptors, as well as the fact that only 39.6% of preceptors invited to participate had indicated a willingness to host a student within the past 12 months. There was potential for a positive response bias with the researchers being known to most preceptor participants.

While the overall response rate for the needs analysis was low, the mixed methods study design with a participatory approach provided breadth and depth to the analysis, thus increasing the validity of the findings. This approach was also instrumental in ensuring that the program was able to be tailored to the needs of JCU preceptors. The broad experience of the research team in both teaching and pharmacy practice was advantageous in providing the most appropriate educational design for the program.

It is anticipated that this program will continue to be offered on an annual basis, with refinements being continually undertaken in response to stakeholder feedback. Considering preceptor feedback from previous studies, the wide variation in preceptor experience in both practice and precepting may warrant the streaming of preceptor development programs to cater for both the novice and the more experienced preceptors [5,17].

It is expected that the existing professional requirements for preceptor development will necessitate mandatory training in the future, which will further promote the development of training programs across Australia. While this program is currently focused on training community pharmacy preceptors, there is potential for it to eventually be expanded to include all preceptors across the broad range of experiential placement sites. The general principles of precepting are similar across a range of health professionals and with the increased emphasis on interprofessional practice in healthcare systems, interprofessional training for preceptors may be a practical and cost-effective approach to facilitate preceptor development, with potential for collaboration with other health professional programs such as medicine and nursing [30,51].

Although preference for preceptor development tailored to institutions and the individual preceptor has been identified [4,5], there are notable similarities in key content among documented preceptor development programs. Therefore, consideration could be given in the future to the establishment of a national preceptor development platform or framework, with the flexibility to accommodate the needs of the institution and the preceptor. This has been the experience in Canada, where a prototype for a national online preceptor development program was developed, with the potential for further refinement and implementation across Canada [15,26,46,50]. The continued development of specific competencies for pharmacist preceptors will provide a further underpinning framework for preceptor development programs [14,15,16].

## 5. Conclusions

The training and development of pharmacist preceptors is considered crucial to the success of experiential placements. This study provides an example of a tailored training program which has been informed by the previous literature, as well as through a comprehensive needs analysis and direct consultation with the profession. The development and implementation of this program has been strategically planned and structured to provide preceptor flexibility, with evidence-based and educationally focused content, and linked to a national pharmacist competency framework. Ongoing modifications and extensions to this program in response to program feedback should ensure that it continues to meet the individualized needs of all preceptors. The further development of specific preceptor competencies for pharmacists is recommended to provide and maintain quality and consistency for future preceptor training programs. An increased focus on preceptor development in pharmacy programs will ensure that student placement experiences are of high quality and provide maximum benefit for both the preceptor and the student, promoting the achievement of optimal graduate outcomes.

## Data Availability

This study did not report any data.

## Supplementary Materials

The following supporting information can be downloaded at https://www.mdpi.com/article/10.3390/pharmacy11050148/s1. Figure S1: JCU Pharmacist Preceptor Training Hub Organizational Platform; Table S1: Outline of JCU Online Preceptor Training Program.

## Abbreviations

ACPE: American Council for Pharmacy Education; ADDIE: Analysis, Design, Develop, Implement, Evaluate; APC: Australian Pharmacy Council; APPE: Advanced Pharmacy Practice Experience; CPD: Continuing Professional Development; GP: General Practitioner; IPPE: Introductory Pharmacy Practice Experience; JCU: James Cook University; PSA: Pharmaceutical Society of Australia; SDT: Self-Determination Theory.

## References

[1] The Australian Pharmacy Council Accreditation Standards for Pharmacy Programs 2020. Updated October 2022. https://www.pharmacycouncil.org.au/resources/pharmacy-program-standards/.

[2] Accreditation Council for Pharmacy Education Accreditation Standards and Guidelines for the Professional Program in Pharmacy Leading to the Doctor of Pharmacy Degree 2016. https://www.acpe-accredit.org/pdf/Standards2016FINAL.pdf.

[3] DeAngelis J.T., Wolcott M.D. (2019). A job analysis to define the role of the pharmacy preceptor. Am. J. Pharm. Educ..

[4] O’Sullivan T., Cox C., Darbishire P., Dinkins M., Johanson E., Joseph A., Vos S. (2020). The status and adequacy of preceptor orientation and development programs in US pharmacy schools. Am. J. Pharm. Educ..

[5] Williams C., Wolcott M., Minshew L., Bentley A., Bell L. (2021). A qualitative preceptor development needs assessment to inform program design and effectiveness. Am. J. Pharm. Educ..

[6] Knott G., Mylrea M., Glass B. (2020). A scoping review of pharmacy preceptor training programs. Am. J. Pharm. Educ..

[7] Howard M.L., Yuet W.C., Isaacs A.N. (2020). A review of development initiatives for pharmacy student and resident preceptors. Am. J. Pharm. Educ..

[8] Lucas C., Williams K., Tudball J., Walpola R. (2018). Community, hospital and industry preceptor perceptions of their role in experiential placements—The need for standardization of preceptor responsibilities and evaluations on students. Curr. Pharm. Teach. Learn..

[9] Knott G., Mylrea M., Glass B. (2022). What do preceptors want? A mixed methods study exploring pharmacist preceptor perceptions of their training and support needs at a regional Australian University. Pharm Educ..

[10] Knott G.J., Mylrea M.F., Glass B.D. (2022). Pharmacy student perceptions of the roles and attributes of pharmacist preceptors in Australia. Pharmacy.

[11] Sonthisombat P. (2008). Pharmacy student and preceptor perceptions of preceptor teaching behaviors. Am. J. Pharm. Educ..

[12] O’Sullivan T.A., Lau C., Patel M., Mac C., Krueger J., Danielson J., Weber S. (2015). Student-valued measurable teaching behaviors of award-winning pharmacy preceptors. Am. J. Pharm. Educ..

[13] Young S., Vos S.S., Cantrell M., Shaw R. (2014). Factors associated with students’ perception of preceptor excellence. Am. J. Pharm. Educ..

[14] Bartlett A.D., Um I.S., Luca E.J., Krass I., Schneider C. (2020). Measuring and assessing the competencies of preceptors in health professions: A systematic scoping review. BMC Med. Educ..

[15] Walter S., Mulherin K., Cox C. (2018). A Preceptor competency framework for pharmacists. Part 2 of a 3-part series. Curr. Pharm. Teach. Learn..

[16] Zeitoun A., Sacre H., Hallit S., Zeenny R., Sili G., Salameh P. (2020). Clinical preceptor competencies for a better pharmacy education: A suggested framework for Lebanon. J. Pharm. Policy Pract..

[17] Enderby C.Y., Davis S., Sincak C.A., Shaw B. (2021). Health-system pharmacist preceptor development and educational needs for accessible resources. Curr. Pharm. Teach. Learn..

[18] Spatioti A., Kazanidis I., Pange J. (2022). A comparative study of the ADDIE Instructional Design Model in distance education. Information.

[19] Condrey T. (2015). Implementation of a preceptor training program. J. Contin. Educ. Nurs..

[20] National Competency Standards Framework, 2016. Pharmaceutical Society of Australia. https://www.psa.org.au/wp-content/uploads/2018/06/National-Competency-Standards-Framework-for-Pharmacists-in-Australia-2016-PDF-2mb.pdf.

[21] Orsini C., Evans P., Jerez O. (2015). How to encourage intrinsic motivation in the clinical teaching environment?: A systematic review from the self-determination theory. J. Educ. Eval. Health Prof..

[22] Ramani S., Leinster S. (2008). AMEE Guide no. 34: Teaching in the clinical environment. Med. Teach..

[23] Howie P., Bagnall R. (2013). A critique of the deep and surface approaches to learning model. Teach. High. Educ..

[24] Taylor D.C., Hamdy H. (2013). Adult learning theories: Implications for learning and teaching in medical education: AMEE Guide No. 83. Med. Teach..

[25] Bower D. (2008). Using adult learning concepts to create a positive pharmacy preceptorship experience. Can. Pharm. J..

[26] Mulherin K., Walter S., Cox C. (2018). National preceptor development program (PDP): Influential evidence and theory. The first of a 3-part series. Curr. Pharm. Teach. Learn..

[27] Praslova L. (2010). Adaptation of Kirkpatrick’s four level model of training criteria to assessment of learning outcomes and program evaluation in higher education. Educ. Assess. Eval. Acc..

[28] Burgess A., van Diggele C., Roberts C., Mellis C. (2020). Key tips for teaching in the clinical setting. BMC Med. Educ..

[29] Vos S., Brown M., Cardello M., Dintzner M., MacKinnon G., Maroyka E., Mbi P., Park S., Weaver K., Zeeman J. (2018). The Report of the 2017–2018 Professional Affairs Standing Committee: The development of the preceptor self-assessment tool for entrustable professional activities for new graduates. Am. J. Pharm. Educ..

[30] Mukhalalati B., Awaisu A., Elshami S., Paravattil B., Zolezzi M., Abu-Hijleh M., Moslih-Almoslih A., Carr A., Bawadi H., Romanowki M. (2022). Assessment of educational needs and design of a preceptor development program for health professional education programs in Qatar. J. Contin. Educ. Health Prof..

[31] Smith D.V., Gauntreaux S., Gulbis A.M., Bruno J., Garey K., Roux R.K., Varkey D. (2020). Program development of a preceptor bootcamp for operational pharmacy preceptors. Am. J. Health Syst. Pharm..

[32] Macedo L., Sturpe D., Haines S., Layson-Wolf C., Tofade T., McPherson M. (2015). An objective structured teaching exercise (OSTE) for preceptor development. Curr. Pharm. Teach. Learn..

[33] Le M., Luong S., Mong R., Gruenberg K., Clinard V. (2022). Examining participant perceptions of the UCSF school of pharmacy master preceptor program. Curr. Pharm. Teach. Learn..

[34] Bloomfield J.G., Jones A. (2013). Using e-learning to support clinical skills acquisition: Exploring the experiences and perceptions of graduate first-year pre-registration nursing students—A mixed method study. Nurse Educ. Today.

[35] McKinney W.P., Rodgers K., Uden-Holman T., Alperin M. (2017). Assessing the evidence for the educational efficacy of webinars and related internet-based instruction. Pedagog. Health Promot..

[36] Prithishkumar I., Michael S. (2014). Understanding your student: Using the VARK model. Educ Forum..

[37] Shatto B., Erwin K. (2017). Teaching millennials and generation Z: Bridging the generational divide. Create. Nurs..

[38] Schneider S.R., Soric M., Schneider S., Wisneski S. (2017). The art of teaching. The Effective Pharmacy Preceptor.

[39] Vos S., Trewet C. (2012). A comprehensive approach to preceptor development. Am. J. Pharm. Educ..

[40] Davison K., Bullen K., Ling J. (2018). Pre-registration pharmacist tutor training: A pilot study. Clin. Teach..

[41] McDuffie C., Duke L., Stevenson L., Sheffield M., Fetterman J., Staton A., McCullough E. (2011). Consortium-based approach to an online preceptor development program. Am. J. Pharm. Educ..

[42] Boyle C., Morgan J., Layson-Wolf C., Rodriguez de Bittner M. (2009). Developing and implementing an academy of preceptors. Am. J. Pharm. Educ..

[43] Cerulli J., Briceland L. (2004). A streamlined training program for community pharmacy advanced practice preceptors to enable optimal experiential learning opportunities. Am. J. Pharm. Educ..

[44] Marriott J., Taylor S., Simpson M., Bull R., Galbraith K., Howarth H., Leversha A., Best D., Rose M. (2005). Australian national strategy for pharmacy preceptor education and support. Aust. J. Rural Health.

[45] Duke L.J., Unterwagner W.L., Byrd D.C. (2008). Establishment of a multi-state experiential pharmacy program consortium. Am. J. Pharm. Educ..

[46] Brownfield A., Williams C., Cox C., Davis L., Haines S., Rambaran K., Ruble M., Douglass Smith M. (2021). Moving a national preceptor development platform from design to reality. Am. J. Pharm. Educ..

[47] Tofade T., Kim J., Lebovitz L., Leadon K., Maynor L., Culhane N., Freeberry M., Harris J., Abate M. (2015). Introduction of a continuing professional development tool for preceptors: Lessons learned. J. Pharm. Pract..

[48] Johnston K., O’Reilly C.L., Scholz B., Georgousopoulou E.N., Mitchell I. (2021). Burnout and the challenges facing pharmacists during COVID-19: Results of a national survey. Int. J. Clin. Pharm..

[49] Shah S., Chojnacki R., Meyer J., Margolis A. (2020). Characteristics of individuals who chose to participate in a preceptor continuing professional development program. Pharmacy.

[50] Cox C., Mulherin K., Walter S. (2018). National preceptor development program (PDP) prototype. The third of a 3-part series. Curr. Pharm. Teach. Learn..

[51] Cox C., Samuel N., Cheon J. (2017). Use of an innovative interprofessional mini-series movie to train preceptors. Inov. Pharm..

