# Supplementary material for: How Should We Prepare Our Pharmacist Preceptors? Design, Development and Implementation of a Training Program in a Regional Australian University
Source: Pharmacy (Basel). 2023 Sep 17;11(5):148. doi: 10.3390/pharmacy11050148 (PMC10514868; doi:10.3390/pharmacy11050148)

Supplementary Materials

<u>JCU Pharmacy Preceptor Training - Program Outline</u>

- Welcome to the program
- Module 1 – Program Overview and the preceptor as a ROLE MODEL
- Module 2 – The preceptor as an EDUCATOR
- Module 3 – The preceptor as an ASSESSOR
- Module 4 – The Preceptor as a MENTOR
- Preceptor online small group interactive NETWORKING SESSION
- Preceptor online discussion forum (optional)
- Post-training survey and Certificate of Completion

Table S1: Outline of JCU Online Preceptor Training Program

Figure S1: JCU Pharmacist Preceptor Training Hub Organizational Platform

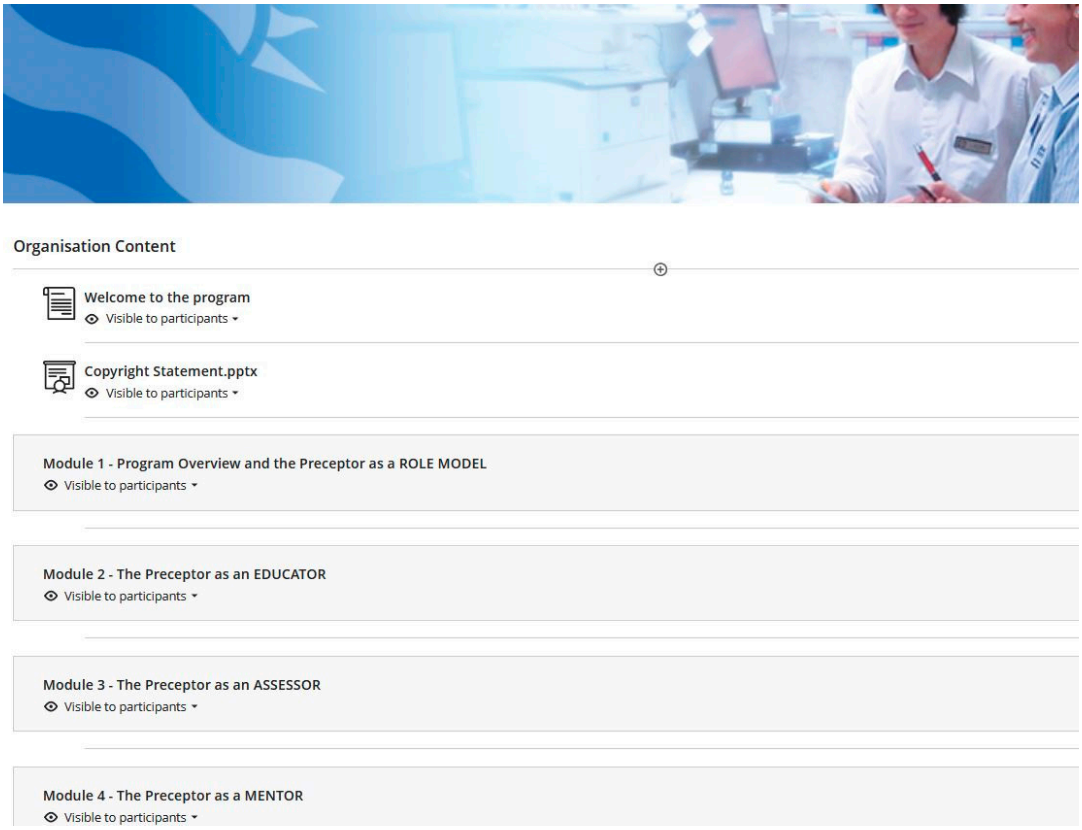

Supplement: Supplementary file 1 [file pharmacy-11-00148-s001.zip › pharmacy-2570366-supplementary.pdf]
